# *In vitro* Impact of Yeast Expressed Hybrid Peptide CATH-2TP5 as a Prophylactic Measure Toward Sepsis and Inflammation

**DOI:** 10.3389/fbioe.2020.00454

**Published:** 2020-06-03

**Authors:** Baseer Ahmad, Quratulain Hanif, Xubiao Wei, Lulu Zhang, Naveed Sabir, Zhongxuan Li, Junhao Cheng, Shahzad Akbar Khan, Abdul Basit, Muhammad Shahid, Amin ur Rehman, Dayong Si, Rijun Zhang

**Affiliations:** ^1^State Key Laboratory of Animal Nutrition and Feed Sciences, Laboratory of Feed Biotechnology, College of Animal Science and Technology, China Agricultural University, Beijing, China; ^2^National Institute for Biotechnology and Genetic Engineering, Faisalabad, Pakistan; ^3^Department of Biotechnology, Pakistan Institute of Engineering and Applied Sciences, Nilore, Pakistan; ^4^College of Veterinary Medicine, China Agricultural University, Beijing, China; ^5^Guangzhou Institute of Biomedicine and Health, Chinese Academy of Sciences, Guangzhou, China; ^6^College of Life Sciences, China Agricultural University, Beijing, China

**Keywords:** cathelicidin, hybrid peptide, endotoxin neutralization, inflammation, apoptosis

## Abstract

CATH-2TP5 is a linear cationic hybrid peptide, consequent from naturally occurring antimicrobial peptide (AMPs) Cathelicidin-2 (CATH-2) and Immunomodulatory peptide Thymopentin (TP5) having dynamic and potent anti-inflammatory activities without hemolytic effect. The biocompatible mechanism of CATH-2TP5 is favored to explore new methodologies in the direction of biomedical applications. In this retrospectively study, an antiendotoxin and anti-inflammatory hybrid peptide CATH-2TP5 was emulated into pPICZα-A and successfully expressed in *Pichia pastoris (P. pastoris)*. The recombinant CATH-2TP5 was purified through the Ni-affinity column and reversed-phase HPLC. The purified CATH-2TP5 peptide exhibited robust anti-endotoxin activity and significantly (*p* < 0.05) neutralized the effect of lipopolysaccharide (LPS). Furthermore, the down-regulated effect of CATH-2TP was more pronounced (*p* < 0.05) on LPS-induced cytotoxic effects, nitric oxide secretion and pro-inflammatory cytokines (TNF-α, IL-6, and IL-1β) in murine RAW264.7 macrophages. As associated to control and parental peptide the number of apoptotic cells was also contracted with the treatment of CATH-2TP5. Thus, we concluded that CATH-2TP5 peptide may be used in various biomedical applications as a therapeutic drug.

## Introduction

Antimicrobial peptides (AMPs) act as an imperative component of the innate immune response in vertebrates and invertebrates against microbial infections (Golec, 2007; Mardirossian et al., 2016). As a major aspect of their defense system, mostly animals produce a variety of peptides, which have both immunomodulatory, and antimicrobial properties (Nikaido, 2003; Mansour et al., 2014). AMPs peptides capable of being applied to treat various microbes as they act on the bacterial membrane through several processes including pore-formating, barrel-stave, and carpet model which were completely different from traditional drugs (Wu et al., 2014). Antibiotic resistance is a major global issue from both an economic and social perspective (Laxminarayan et al., 2013). Despite the accessibility of drugs and antibiotics, microbial infections are the quite main cause of death due to the incapability of the particular therapeutics to kill microbes and bind their pathogenic effect like LPS (Lu et al., 2010; Hwang et al., 2013). Gram-negative bacteria contain LPS in the outer leaflet of membrane that usually plays a key role in the pathogenesis of respiratory diseases, chronic illness and septic shock (Golec, 2007; Pompilio et al., 2012; Mardirossian et al., 2016). Gram-negative bacteria release LPS which is recognized by toll-like receptor 4 (TLR4) on macrophages ultimately triggering the activation of cytokines that ultimately leads to inflammation and sepsis (Nagaoka et al., 2001). AMPs have been determined as a potential source in applied medicine as antibacterial, immunomodulatory and anti-inflammatory agents. Accordingly, there is an instantaneous and urgent need for the development of new peptides that have both antibacterial and LPS neutralizing activities.

Cathelicidin-2 (CATH-2) is a highly effective cationic peptide that is under investigation for its potential therapeutic use against bacterial and fungal infections. CATH-2 inhibits LPS-induced inflammatory cytokines production such as tumor necrosis factor-alpha (TNF-α), interleukin-6 (IL-6), interleukin-1β (IL-1β) and induces expression of immune cells (Solmajer, 1990; Bals et al., 1999; Pompilio et al., 2011; Hancock et al., 2016). Systemic immune activation by LPS can be lethal, so inhibition of inflammatory reactions during sepsis is essential to protect the host from over-secretion. The other peptide Thymopentin (TP5) includes the amino acids (Arg–Lys–Asp–Val–Tyr) and represents residues 32–36 of the nuclear protein thymopoietin (Goldstein et al., 1979). TP5 shows likewise organic action as thymopoietin in-charge of phenotypic separation of T-cells and the regulation of the immune system (Singh et al., 1998). It has been perceived as an immune modulator for the treatment of essential immune deficiency diseases such as AIDS (Sundal and Bertelletti, 1994), rheumatoid arthritis (Shah et al., 2017), and immune system infections.

The application of naturally occurring AMPs displays higher nephrotoxicity, promoting hemolysis, toxicity, and neurotoxicity (Cereghino and Cregg, 2000). Subsequently, it is challenged to develop synthetic peptide based on naturally occurring AMPs to successfully treat infectious diseases without its harmful effects. However, hybridizing different peptides have taken rising attention to the development of new agents having antibacterial, anti-inflammatory properties with less cytotoxicity (Solmajer, 1990; Pompilio et al., 2011). Furthermore, a cost-effective and active production-based method is required for commercialization of hybrid peptides whereas, due to the high expense of peptide synthesis the heterologous expression of the recombinant peptide is the best choice. Methylotrophic yeast expression system has been an excellent host for the large-scale production of the hybrid peptides (Sreekrishna et al., 1997). The advantages to this system include the stable integration into the expression plasmid at a specific site, extra-cellular secretion of the recombinant protein into the medium, and a simple purification procedure of secreted proteins (Sreekrishna et al., 1997). Therefore, several anti-microbial peptides have been produced on a large scale and have been successfully expressed in *P. pastoris* (Li et al., 2005; Jin et al., 2006b).

Moreover, our previous laboratory designed and expressed hybrid peptide LL-37Tα1 in *P. pastoris* depicted its immunomodulatory, anti-inflammatory, and antiendotoxin activities (Ahmad et al., 2019). In the current study, we sought to assess that the combination of CATH-2 (16 amino acid) and Thymopentin (5 amino acid) may have improved endotoxin binding, anti-inflammatory activity along with tiniest cytotoxic effects. Consequently, we synthesized and expressed the hybrid peptide CATH-2TP5 in methylotrophic yeast and explored its biological activities.

## Materials and Methods

### Strains, Vectors, and Reagents

The TIANprep plasmid extraction kit was obtained from Taingen biotech, Beijing, China. The restriction enzymes *Kpn*I, *Xba*I, and *Sac*I were purchased from TaKaRa Biotechnology (Dalian, China). Protein marker (Thermo Fisher Scientific, United States), Zeocin (Invitrogen, Carlsbad, CA, United States). *P. pastoris* (*strain X-33*), *E. coli* (*strain DH5*α), expression vectors pPICZαA and lipopolysaccharides (LPS) from *Escherichia coli* O55: B5 were obtained from (Sigma, Aldrich, United States) and have been routinely used in our lab.

### Growth Media

The *E. coli* (*DH5*α) strain was cultured in Luria-Bertani (LB) medium (5 g/L yeast extract, 10 g/L tryptone, and 10 g/L NaCl). Yeast extract peptone dextrose (YPD) medium (2% peptone, 1% extract yeast, 2% dextrose, and 1 M sorbitol) was used as growth medium for the growth of *P. pastoris* (X-33). The transgenic *P. pastoris* (X-33) strains were cultivated in minimal dextrose, buffered complex glycerol (BMGY) medium (2% tryptone, 1% yeast extract, 1.34% YNB, 4 × 10^–5^% biotin, 1% glycerol, and 100 mM potassium phosphate, pH 6.0). The recombinant CATH-2TP5 was incubated in buffered complex methanol (BMMY) medium (2% peptone, 1% yeast extract, 100 mM potassium phosphate, 1.34% YNB, 4 × 10^–5^% biotin and methanol, pH 6.0).

### Cloning and Expression of the Recombinant Plasmid

The amino acid sequence of the hybrid CATH-2TP5 peptide was optimized through the JAVA codon adaptation tool (JCAT)^1^. The full-length gene of 96 bp was synthesized by Sangon Biotech and expressed in vector pPICZαA at the restriction sites of *Kpnl* and *Xba*I with 6 × Histidine tag (His-tag). The transformation of expressed plasmid was carried out in *E. coli strain DH5*α. The positive transformant colonies were selected on low-salt LB plates and confirmed by PCR using specific primers as P00-1 F: 5′-CCAGATGGGGTAGATTCTT-3′ P00-2 R 5′-GTAACGTCCTTTCTCTTTG-3′ and then sequenced (Tsingke Biotech). The reaction mixture was incubated at 95°C for 5 min, followed by denaturation for 30 s at 95°C, annealing at 52°C for 30 s, extension at 72°C for 50 s and a final extension at 72°C for 10 min. The efficacious recombinant strains were then cultured overnight and the plasmid was attained. The *Sac*I linearized pPICZαA plasmids were transmuted into *P. pastoris strain* (*X-33*) by electroporation method as specified by the *Pichia* expression guide. The positive cells were further selected on YPD agar plates containing antibiotics (Zeocin 100 μg/mL). The addition of the aim genes into the *P. pastoris* genome was confirmed by PCR using both (sense and antisense) specific primers. For negative control, a vacant expression vector pPICZαA was prepared. The confident colonies of *P. pastoris* were cultured in 5 mL tubes of BMGY medium at 28°C, 200 rpm for 12 h until the OD_600_ reached to 4–6. After centrifugation (at 3,000 × *g*) for 5 min and the collected cells were resuspended in 50 mL of BMMY containing 1.34% YNB and biotin (4.0 μg/mL) in a 250 mL shake flask and incubated at 28°C. Pure methanol was consequently added to the culture medium at the final concentration of 1% every 24 h to maintain induction (Ausubel et al., 1991; Ahmad et al., 2019). All induction experiments were performed in triplicate.

### Purification and Mass Spectrometry Analysis of the Recombinant CATH-2TP5

The expressed recombinant CATH-2TP5 peptide was purified after the removal of cell debris from culture supernatant via centrifugation at 12,000 rpm for 20 min at 4°C. The supernatant (containing CATH-2TP5 peptide with 6 × His-tag) was sieved and laden to 1 ml His Trap Chelating Ni-affinity column (Bio-Beads TM, Sweden). The column was equilibrated with 1 × phosphate buffer (PB) and 10 mM imidazole. The adsorbed hybrid recombinant peptide was extracted by using various concentrations of a linear gradient of imidazole (50–500 mM). The eluted samples were sized-separated by Tricine-SDS-PAGE. The Bio-Rad dye agent with bovine serum albumin (BSA) was used as a standard for the evaluation of protein concentration (Ausubel et al., 1991). After early purification, the peptide was additional purified by using reverse-phase high-performance liquid chromatography (RP-HPLC) on a Kromasil C18 column (4.6 × 250 mm, 5 μm) with a linear gradient of acetonitrile (0–100% for 30 min) containing 0.1% Trifluoroacetic acid (TFA) at a flow rate of 1.0 ml/min. The elution points were detected at 220 nm and the active fraction was verified by using quantitative Chromogenic limulus amebocyte lysate (LAL) (Ahmad et al., 2019). The RP-HPLC purified CATH-2TP5 was diluted with Milli-Q water and filtered through 0.22 μm and further subjected to electrospray ionization mass spectrometry ESI-MS/MS.

### Activity Assay of CATH-2TP5

#### Lipopolysaccharide (LPS) Neutralization Assay

The endotoxin neutralization of parental (CATH-2) and hybrid (CATH-2TP5) peptide were assessed using a Chromogenic limulus amebocyte lysate (LAL) according to manual instruction. Different concentrations of parental and hybrid peptides (15 to 40 μg/mL) were treated with a constant concentration of LPS (1 EU/mL) at 37°C for 15 min in the wells of a pyrogenic sterile microliter plate. Furthermore, 50 μL of the aliquots were then supplemented to equal volumes of LAL reagent and incubated for 6 min at 37°C. A reaction was suspended with the addition of HCl solution and analyzed on OD_545_ nm (Ahmad et al., 2019).

#### Hemolytic Activity Assay

Mouse red blood cells isolated from heparinized blood as described previously (Ahmad et al., 2019) were used to analyze the hemolytic activity of parental and hybrid peptides. A 4 ml mice RBCs were centrifuged at 1,500 rpm for 10 min. The cells were washed three times with diluted 10% hematocrit and incubated with various concentrations (15 to 40 μL) of hybrid CATH-2TP5 peptide for 1 h at 37°C. The sample was further centrifuged at 3,500 rpm for 5 min and absorbance of the supernatant was analyzed on 414 nm.

#### Cell Culture

Dulbecco’s Modified Eagle Medium (DMEM) was used to cultured murine macrophage (RAW264.7) along with antibiotics (100 μg/mL) streptomycin, 100 U/mL penicillin) and 10% fetal calf serum (HyClone, Thermo Fisher Scientific, United States) in a humidified chamber at 37°C under 5% CO_2_.

#### LPS-Induced Cytotoxicity Detection by Lactate Dehydrogenase Activity (LDH) Assay

Lactate dehydrogenase activity assay was used to assess the cytotoxic effect of hybrid peptide on murine RAW264.7 macrophages infected with or without LPS (1 μg/mL) incubated at 24 h as described previously (Anton et al., 2012; Ghonime et al., 2014). Additionally, LPS-induced cells (1 × 10^5^ cells/mL) were exposed to parental and hybrid peptide (15 to 40 μg/mL) to plaid the comparative effect. The supernatants were collected and the cytotoxic level was measured according to the kit instructions (Dojingdo Laboratories, Kumamoto, Japan).

#### Quantification of Nitric Oxide (NO) and Pro-inflammatory Cytokines Production LPS-Induced Mouse RAW264.7 Macrophages

Mouse RAW264.7 cells (5 × 10^5^/well) were incubated with (1 μg/mL) LPS in the presence and absence of parental and hybrid (15 to 40 μg/mL) peptide in DEME medium in a humidified chamber with 5% CO_2_ atmosphere. The culture supernatant was assorted with Griess reagent and incubated for 10 min at room temperature. The secretion of NO was measured on a microplate reader at 540 nm (Green et al., 1982).

The effect of parental and hybrid peptide on the expression of TNF-α, IL-6, and IL-1β in LPS-stimulated mouse RAW 264.7 macrophages was quantified by enzyme-linked immunosorbent assay (ELISA; Cloud-clone corp, Houston, United States) as described previously (Ahmad et al., 2019).

#### Determination of Apoptosis via Flow Cytometry

Lactate dehydrogenase activity-induced apoptosis in RAW264.7 macrophages was interpreted by annexin V-FITC (V conjugated to green-fluorescent FITC dye) and PI (Propidium iodide) staining procedure. The mouse RAW264.7 macrophages (1 × 10^5^ cells/mL) were cultured as described above. The cultivated cells were infected with LPS (10 μg/mL) in the presence and absence of parental and hybrid peptides (40 μg/mL) for 4, 12, and 24 h. After treatment, the cells were washed thrice with ice-cold PBS (washing buffer) and stained with FITC annexin V/PI apoptosis detection kit (Becton Dickinson, Franklin Lakes, NJ, United States) as described previously (Shahid et al., 2017; Ahmad et al., 2019). Apoptosis rate was expressed using a FACSA by flow Cytometry (Becton Dickinson, United States).

### Statistical Analysis

The three independent experiment data were expressed as mean ± standard deviation (SD). Statistical data were estimated by the one-way analysis of variance (ANOVA) according to the least significant difference (LSD) test for multiple comparisons. A *post hoc* test using SPSS 19.0 (SPSS, Inc., Chicago, IL, United States). For all analysis, *p* < 0.05 and *p* < 0.01 were considered significant.

## Results

### Optimization and Expression of the Recombinant CATH-2TP5

**FIGURE 1:**
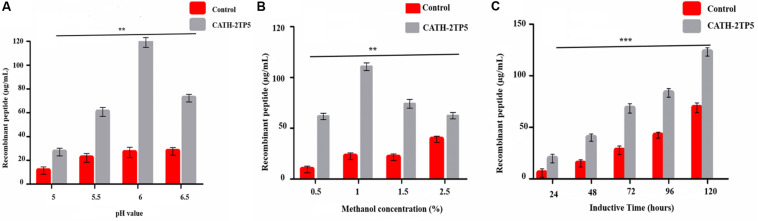
Effects of pH value, methanol concentration, and inductive time and inductive on the peptide expression. **(A)** Recombinant peptide expression was conducted at different pH values (pH 5-6.5), induced by 1% final methanol concentration at 28°C for 120 h. **(B)** Recombinant peptide expression was conducted at pH 6, inducted by various methanol concentration (0.5–2.5%) at 28°C for 120 h. **(C)** Recombinant peptide expression was conducted at pH 6.0, inducted by 1% methanol concentration at 28°C for 24–120 h. Data are presented as mean ± SD, *n* = 3. While **p < 0.01, ***p < 0.001 vs. Controls.

Yeast expression vector pPICZαA-CATH-2TP5 that contains a 96 bp DNA fragment encoding a recombinant C-terminal 6 × His-tagged that facilitate purification and analysis of expressed proteins. The restriction enzyme *Kpn*I and *Xba*I were attached at its 5′ and 3′ ends and fragment was cloned in the frame of α-factor signal, downstream promoter alcohol oxidase (AOXI) of the expression vector pPICZαA and the result in recombinant pPICZαA-CATH-2TP5 after double digested with a restriction enzyme (Supplementary Figure S1). The adjustment of the inserted peptide sequence was confirmed by PCR. The recombinant plasmid pPICZαA-CATH-2TP5 was linearized with *Sac*I and altered into the competent cells of *P. pastoris* (*X-33*) by electroporation. The zeocin (100 μg/mL) resistant *P. pastoris* transformants were obtained and further subjected to PCR and sequencing for the confirmation of positive transformants. Our result exhibited that pPICZαA-CATH-2TP5 was successfully assimilated into host cells. Moreover, the expression conditions were optimized according to the method described above. The optimized factors included initial pH value (pH 5-6), final methanol concentration (0.25–2.0%) and incubation time (24–120 h). The optimal conditions for recombinant peptide expression were pH 6, and incubation for 120 h with 1% methanol. The expression level of recombinant CATH-2TP5 peptide was 119, 110, and 123 μg/ml, respectively (Figures 1A–C).

**FIGURE 2:**
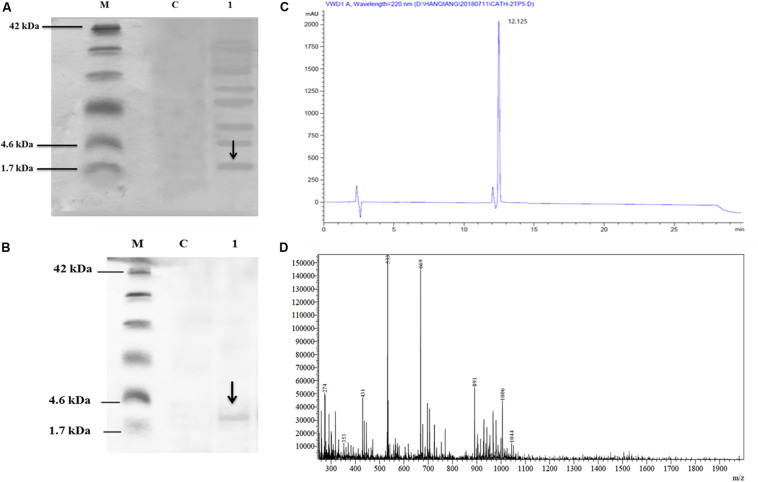
Recombinant hybrid peptide CATH-2TP5 analysis, **(A)** Tricine-SDS-PAGE of yeast expressing CATH-2TP5 medium. Molecular mass markers (Lane M); Control PpICZαA-blank (Lane C); Supernatant PpICZαA-CATH-2TP5, separated by 15% Tricine-SDS-PAGE with sliver stained and arrow showed peptide (Lane 1). **(B)** Tricine-SDS-PAGE of the purified peptide. Molecular weight markers (Lane M); Control PpICZαA-blank (Lane C). A sample of the purified peptide and arrow specified CATH-2TP5 2.6 kDa (Lane 1). **(C)** RP-HPLC of hybrid peptide and high peak exhibited fraction that contains CATH-2TP5. **(D)** ESI-MS of purified hybrid recombinant CATH-2TP5 peptide.

The culture supernatant was separated on Tricine-SDS-PAGE and silver staining. The expression of the recombinant CATH-2TP5 in *P. pastoris* indicated the apparent molecular weights of 2.6 kDa (Figure 2A).

### Purification and Mass Spectrometry Analysis of Recombinant CATH-2TP5

After centrifugation of the cultured media, supernatant was purified by Ni-NTA affinity chromatography column. The pure CATH-2TP5 peptide was eluted with 500 mM imidazole. As shown in Figure 2B, SDS-PAGE of the recombinant purified peptide appeared as a single band with molecular weight of 2.6 kDa. The peptide was further applied to RP-HPLC and resultant product was highly purified (yielded 4 mg) which presented in Figure 2C. Furthermore, the purified recombinant CATH-2TP5 was diluted in milli-Q water, filtered and analyzed by ESI-MS/MS. Mass spectrometry of the purified CATH-2TP5 expressed a single non-dispersed signal (Figure 2D). The average weight of the molecular ion [M+5H+]5+ is 533 Da, [M+4H+]4+ was 669 Da, and [M+3H+]3+ is 891 which parallels to the molecular mass of 2670.62 Da for the recombinant CATH-2TP5. Our result revealed that hybrid recombinant peptide was removed from C-terminus successfully.

### Anti-inflammatory and Immunomodulatory Activities

#### CATH-2TP5 Neutralizes Lipopolysaccharide (LPS)

**FIGURE 3:**
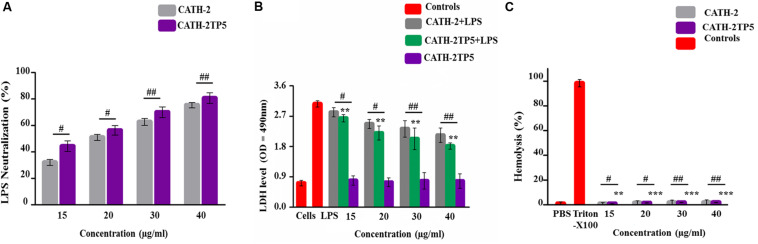
Endotoxin neutralization, cytotoxicity, and hemolytic activity test of parental and recombinant CATH-2TP5 peptide. **(A)** LPS neutralization by CATH-2 and CATH-2TP5 indomitable by using an endotoxin quantitation kit. Mean values accessible; *n* = 3 ± SD (^#^*p* < 0.05 and ^##^*p* < 0.01 revealed contrast of CATH-2 vs. CATH-2TP5). **(B)** CATH-2TP5 diminished cytotoxicity in the supernatant of endotoxin-infected murine RAW264.7 macrophages. Data signified as mean ± standard deviation (SDs) of independent experiments. **p < 0.01 vs. LPS. While, ^#^*p* < 0.05 and ^##^*p* < 0.01 specifies significant variance related with parental CATH-2 peptide. **(C)** Hemolytic activities of CATH-2TP5 in contradiction of mouse RBCs. The data resemble to the mean values of three independent experiments and are expressed as a % age of hemolysis ± standard deviation (**p < 0.01, ***p < 0.001 vs. Triton X-100). Whereas, ^#^*p* < 0.05 and ^##^*p* < 0.01 indicates significant difference compared with parental CATH-2 peptide.

LAL test was used to asses the aptitude of parental and hybrid peptide to neutralize the LPS. The CATH-2TP5 peptide has a net charge of + 9 under the physiological conditions and it can neutralize and bind with LPS. Our results exhibited that CATH-2 (35 and 40 μg/ml) was able to neutralize LPS (62.321% ± 2.321, 75.331% ± 2.456, respectively) and CATH-2TP5 peptide (70.656% ± 4.382, 80.1% ± 4.395, respectively) in a dose-dependent manner. However, as compared to parental peptide, neutralization of LPS by hybrid peptide was more pronounced (*p* < 0.05) (Figure 3A).

#### CATH-2TP5 Ameliorates the Cytotoxic Effects of LPS in Mouse RAW264.7 Macrophages

Lactate dehydrogenase activity release into cell medium was used as an indicator of cell death with the NAD^+^ reduction. The cells along with only LPS infection showed higher level of LDH at 24 h (3.06 ± 0.067) as associated with co-infection of LPS plus parental and hybrid peptide (15 to 40 μg/mL), and control group. This describes that LPS significantly (*p* < 0.05) damaged the mouse RAW264.7 macrophages. However, the LPS-induced cytotoxicity significantly (*p* < 0.01) decreased 2.3 ± 0.077 to 2.03 ± 0.067, respectively with a concentration of LPS (35 μg/mL), while the cytotoxic effect of LPS at a concentration of 40 μg/mL lowered from 2.13 ± 0.048 to 1.81 ± 0.032, respectively when cells were treated with CATH-2 and CATH-2TP5 peptides. Furthermore, CATH-2TP5 decreased the LDH level as compared with CATH-2. These results elaborated that CATH-2TP5 peptide neutralizes the LPS and significantly lowered the cytotoxic effects of LPS in a dose dependent manner (Figure 3B).

The hybrid CATH-2TP5 peptide exhibited pronounced anti-endotoxin activity. The hemolytic activity of the parental and recombinant peptide was detected by lysing mouse red blood cells (Figure 3C). Comparatively, with control cells 0% (*p* < 0.001) hemolysis was observed in treated cells and hybrid peptide triggered less hemolysis than CATH-2. Conversely, Our result exhibited that recombinant hybrid CATH-2TP5 peptide does not have cytotoxic and hemolytic activities.

#### CATH-2TP5 Down-Regulates LPS-Induced Inflammatory Response of RAW264.7 Macrophages

**FIGURE 4:**
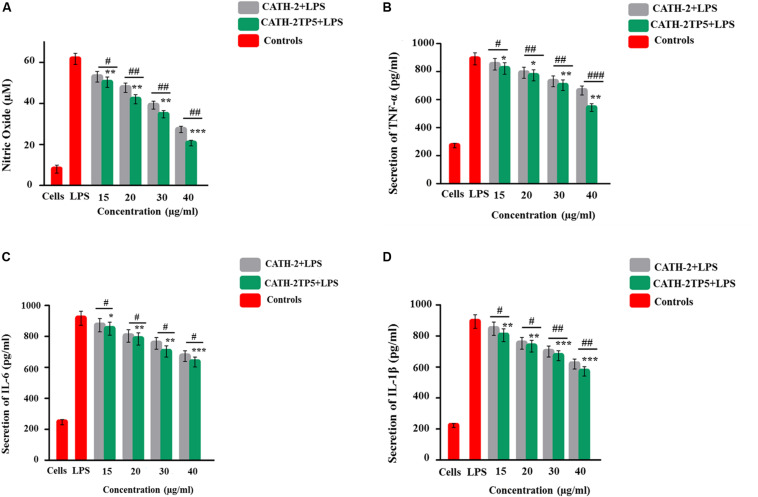
Anti-inflammatory effects of parental and novel CATH-2TP5 peptides on LPS-influenced murine RAW264.7 macrophages. Production of **(A)** NO, **(B)** TNF-α, **(C)** IL-6, and **(D)** IL-1β. Cells were cured with various doses of CATH-2 and CATH-2TP5 peptides for 1 h before the treatment of cells with LPS to triggered infection. After a 24 h incubation, the supernatant was collected, and inflammatory cytokines levels were detected. Values are means ± SDs of three determinations. *p < 0.05, **p < 0.01, and ***p < 0.001 vs. LPS. While, ^#^*p* < 0.05, ^##^*p* < 0.01 and ^###^*p* < 0.001 designates significant variance compared with parental CATH-2 peptide.

The ability of CATH-2TP5 peptide to neutralize LPS provoked us whether CATH-2TP5 could conquest the inflammatory response elicited by LPS. To further investigate the query, we measured the effect of recombinant CATH-2TP5 on LPS-stimulated secretion of NO, and proinflammatory cytokines TNFα, IL-6, and IL-1β from mouse RAW 264.7 macrophages. As shown by the results in Figure 4A, LPS significantly increased the NO secretion compared with control cells (61 μM vs. 8 μM, respectively), but the response was most efficiently inhibited (34 and 21 μM) after treatment with (35 and 40 μg/mL) CATH-2TP5. Moreover, the expression of TNF-α, IL-6, and IL-1β significantly (*p* < 0.05) increased in LPS-stimulated mouse RAW 264.7 cells as compared to control group (Figures 4B–D). Whereas, the treatment with hybrid CATH-2TP5 peptide ceased this increase in a dose-dependent manner. The TNF-α secretion level was significantly (*p* < 0.001) reduced to 703 and 575 pg/mL at a CATH-2TP5 peptide concentration of 35 and 40 μg/mL, respectively (Figure 4B). IL-6 level was also increased with only LPS treatment (915.56 pg/mL) but in the CATH-2TP5 peptide-treated group, the peptide concentration of 35 and 40 μg/mL decreased IL-6 secretion to 725 and 635 pg/mL, respectively (*p* < 0.001) (Figure 4C). Similarly, CATH-2TP5 peptide also inhibited the production of IL-1β to 673 and 568 pg/mL at the peptide concentration of 35 and 40 μg/mL as compared to LPS-induced (895 pg/ml) mouse RAW 264.7 cells, respectively (Figure 4D). These results provide evidence that hybrid CATH-2TP5 peptide has potent anti-inflammatory activity. Furthermore, as compared to parental CATH-2 peptide the hybrid CATH-2TP5 peptide exhibited more anti-inflammatory activities (Figures 4A–D).

#### CATH-2TP5 Reduces LPS-Induced Apoptosis in Murine RAW264.7 Macrophages

**FIGURE 5:**
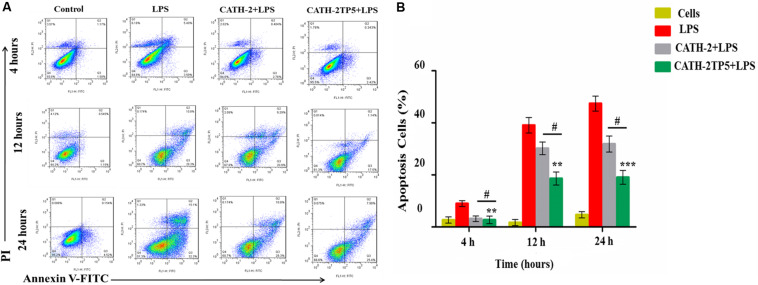
Apoptosis of LPS-induced murine RAW264.7 macrophage at 4, 12, and 24 h with FITC-conjugated annexin-V (Green) and PI (Red). **(A)** The marked RAW264.7 macrophage was observed and analyzed by flow cytometry, the control signifies normal cells, the middle panel LPS discloses demonstrative RAW264.7 macrophage treated with LPS only, and the lower panel reveals RAW264.7 macrophages treated with LPS plus parental and hybrid peptides. **(B)** Observe the effects of LPS with CATH-2TP5 in RAW264.7 macrophages apoptosis. and **p < 0.01, ***p < 0.001 indicate the significance and highly significance difference vs. LPS. While ^#^*p* < 0.05 indicated significance divulges between parental and hybrid peptide.

To explore the role of CATH-2 and CATH-2TP5 peptide in LPS-stimulated apoptosis, mouse RAW264.7 macrophages were cultivated. After 95% of confluency, macrophages were treated with only LPS and LPS plus CATH-5TP5 peptide for 4, 12, and 24 h. the macrophages were stained with annexin V-FITC and PI as per instructions of the manual. The number of apoptotic cells was significantly increased in LPS treated group in both early (annexin V-positive cells) and late-stage (annexin V-and PI-double positive cells) at 4, 12, and 24 h post-infection (Figure 5A). The percentage of apoptotic cells was increased in a time dependent manner 8.99% ± 3%, 39.1% ± 11% and 47.3% ± 13% upon LPS infection after 4, 12, and 24 h, respectively. However, the addition of CATH-2TP5 along with LPS infection significantly reduced (*p* < 0.01) the number of apoptotic cells as compared to LPS infection alone (Figure 5B). Our results provide evidence that CATH-2TP5 peptide could neutralize the LPS and ultimately reduces the LPS-induced apoptosis in RAW 264.7 macrophages.

## Discussion

Hybridization of the natural peptides isolated from different sources is a common technique to construct novel peptides against the pathogenic microbes exhibiting antimicrobial resistance (Bulet et al., 2004; Jenner et al., 2019). AMPs have a positive net charge that allows them to interconnect with a bacterial membrane which has a negative charge, help to bind with endotoxin (Nagaoka et al., 2001). LPS, also known as endotoxin which is released during lysis of Garm-negative bacteria and plays a key role in pathogenesis of endotoxin shock (Nagaoka et al., 2001). In the previous study, the experts have struggled to amend the amino acid sequence of the parental peptides to enhance the expression and activities against infections. Moreover, the well-preserved amino acid sequence of the peptides has an inordinate impact on immunity and anti-inflammatory activities and also the proper replacement or addition in the peptide sequence does not affect its function but also improve the response of hybrid peptides (Liu et al., 2018). However, the development of the novel peptides through hybridizing of various parental peptides is an operative method to enhance the high immunomodulatory and anti-inflammatory activities along with LPS neutralizing ability with least adverse effects (Jang et al., 2006; Lee et al., 2016). Therefore, more attention has been focused on the expression of hybrid peptides with enhanced therapeutic efficacy as it could be beneficial in the treatment of several infectious diseases. The intention of hybrid peptides is based on the amino acid sequence of the parental AMPs such as cecropin, cathelicidin, magainin II, LL37, and melittin (Lee et al., 2002; Jin et al., 2006a; Wu et al., 2014).

There are some methods for the manufacture of hybrid peptides: (i) extraction from natural resources, (ii) chemical synthesis, and (iii) expression in yeast or bacteria (Van Harten et al., 2018). Due to the high expense, the yeast expression system delivers an opportunity for the production of hybrid peptides on a commercial scale. According to the best of our knowledge, we for the first time have optimized the expression conditions like temperature, PH, and concentration of methanol induction in culturing medium of the novel hybrid peptide (CATH-2TP5) in *p. pastoris.* We expressed anti-inflammatory hybrid peptide with the optimum condition. The concentration of recombinant CATH-2TP5 peptide was a maximum of 50 mg/L in the culture medium. Our SDS-PAGE results showed that the size of the recombinant peptide is 2.6 kDa. The expression yield is higher than earlier described such as LL-37Tα1 (Ahmad et al., 2019), ceropinAD (Parachin et al., 2012), and CA-MA (Jin et al., 2006a). In the current study, we also reported the benefits of expression *in P. pastoris* as compared to bacterial expression system concerning cost-effectiveness and heterologous expression of novel active CATH-2TP5 for commercial usage. There are many advantages of a eukaryotic expression over bacterial expression systems such as protein glycosylation, post-translational modifications, folding and processing which is probably absent in the *E.coli* system (Gupta and Shukla, 2017). Comparatively, the bacterial expression system is difficult for the recovery of the small peptides to be recovered so this system could not be used directly to express the peptide with the toxicity to bacteria (Parachin et al., 2012). For pure hybrid peptide, we performed the purification method as described before with slight modification (Jin et al., 2009). For the two steps purification, we used Ni-NTA affinity chromatography column (Zhang et al., 2015) and HPLC, and we attained 4 mg quite pure recombinant CATH-2TP5 peptide from 200 ml culture medium.

In the current study, purified CATH-2TP5 peptide was exposed to endotoxin binding, cytotoxicity, and hemolytic activity reflected as imperative advantages of recombinant peptide to be used as capable drugs. LPS comprises three parts; lipid A, O-antigen and polysaccharide core. Lipid A is unambiguously a physiologically active component of endotoxin and principally involved in stimulating the inflammatory response (Iwanaga et al., 1992). Lipid A is also accountable for the instigation of LAL reagent (Gutsmann et al., 2010a). In the present study, LAL depicted that CATH-2TP5 peptide binds to lipid A part of LPS and neutralizes the biological effects of endotoxin. However, based on previous reports, positive charge and hydrophobicity are essential for the peptide to have LPS neutralizing and anti-inflammatory characteristics (Sun and Shang, 2015; Zhang et al., 2018). In the current study, Our hybrid peptide comprises an N-terminal region along with polar active part of CATH-2 (16 amino acid) and a C-terminal TP5 having a net charge of +9 with reducing hydrophobicity as reported in the previous study (Gutsmann et al., 2010b; Kaconis et al., 2011; Ahmad et al., 2019). It sustained our assumption that the amalgamation of active parts of dissimilar parental peptides conferred strong electrostatic interactions between AMPs and LPS. AMPs have revealed cytotoxic and hemolytic effects on mammalian cells (Zhang et al., 1999; Kaconis et al., 2011; Lee et al., 2016). In the current study, our hybrid peptide showed less cytotoxicity and hemolytic activity as compared with parent peptide due to reduced hydrophobicity which in line with the earlier study (Ahmad et al., 2019).

In the present study, we also detected LPS-induced production of NO, TNF-α, IL-6, and IL-1β in mouse RAW264.7 macrophages. LPS activates macrophages to release NO and potent inflammatory mediators which involved in several acute and chronic diseases containing hemorrhagic shock, endotoxin shock and atherosclerosis (Bertolini et al., 2001; Lind, 2003). Approximately, antibiotics are recognized to kill the bacteria but at the same time they also stimulate the release of LPS and may aggravate the endotoxic shock (Nagaoka et al., 2001) owing unable to neutralize the LPS. So, a drug that subdues the creation of NO, inflammatory cytokines and could neutralize the LPS may be a sensible additional therapy for endotoxin shock. Consequently, preventing these inflammatory cytokines may be potentially effective for averting inflammatory diseases. As compared with previously reported recombinant peptides lunasin-4 (Zhu et al., 2018) and SPHF1 (Ahn et al., 2012), we originate that CATH-2TP5 more proficiently inhibits the production of pro-inflammatory cytokines. These features predict that CATH-2TP5 peptide is an attractive drug candidate for the treatment of inflammation and endotoxin shock produced by Gram-negative bacterial infection.

**FIGURE 6:**
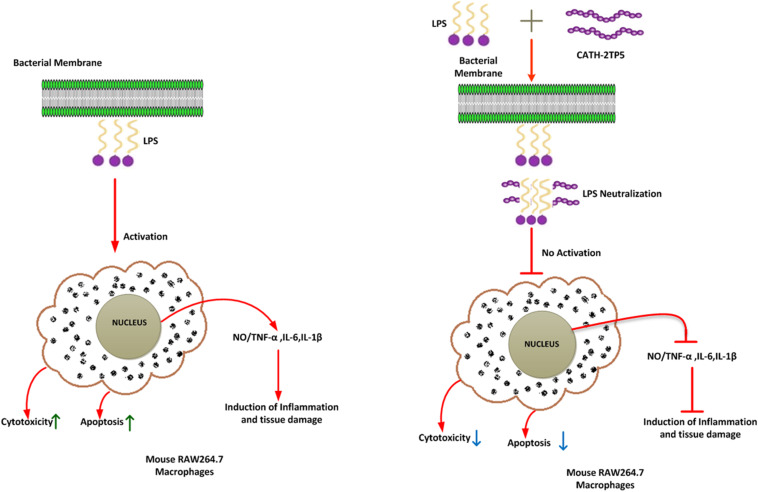
A schematic diagram of the possible mechanism by which CATH-2TP5 counteract endotoxin replies in mouse RAW264.7 macrophages. Left, LPS only; right LPS plus CATH-2TP5. The CATH-2TP5 binds with LPS and neutralizing it. ↑, Activation; ⊣, no activation; ↑, upper regulate responses; ↓, lower regulate responses.

Our findings implicate that CATH-2TP5 treatment effectively reduces the endotoxins and proinflammatory mediator, thus it might have potency for inhibiting inflammatory diseases. Furthermore, our recombinant hybrid peptide depicted high immunomodulatory and anti-inflammatory activities, and also neutralized the bioactivity of endotoxin without cytotoxic and hemolytic effects as modeled in Figure 6. These data are in agreement with the previous study reporting high anti-inflammatory and LPS neutralizing activity of CATH-2 without hemolytic activity (van Dijk et al., 2016; Banaschewski et al., 2017). Moreover, endotoxin up-regulates adhesion molecules (Lush et al., 2000) and induces apoptosis (Bannerman and Goldblum, 2003). In the present study, we used PI and annexin-V FITC staining which expounded that LPS significantly induces apoptosis in murine RAW264.7 macrophages. Under some circumstances, macrophages are responsible for severe systemic inflammatory response triggered by LPS which has deleterious effects and leads to septic shock (Bone, 1991; Guo et al., 2019). We identified that CATH-2TP5 neutralizes LPS and reduces both early and late apoptosis. Generally, these investigations designate that CATH-2TP5 is a propitious peptide that could be a potential candidate for application in the medical industry as therapeutic condidate.

## Conclusion

For the first time, we report a successful expression method for the hybrid CATH-2TP5 peptide in *P. pastoris* with expression plasmid PpICZαA. CATH-2TP5 potently neutralized LPS with no cytotoxic and hemolytic activity. Additionally, CATH-2TP5 novel peptide exhibited immunomodulatory, and anti-inflammatory activity by preventing cytokines release including NO, TNF-α, IL-6, and IL1β, and also decreased the number of apoptotic cells in LPS-infected RAW264.7 macrophages. These results deliver a potential strategy for recombinant production of bioactive CATH-2TP5 in the industry and might help prevent inflammatory diseases.

## Data Availability Statement

All datasets generated for this study are included in the article/Supplementary Material.

## Author Contributions

BA wrote the manuscript. BA, QH, and AB accomplished the trials. BA, QH, and RZ conceived and planned the experiments. BA and QH analyzed the data. BA, QH, NS, and MS contributed to the review and English proficiency. ZL, XW, LZ, SK, AR, and JC contributed to English grammar check. DS and RZ guided and supported the experiments. RZ contributed to the supervision. All authors abetted to read and sanctioned the final manuscript.

## Conflict of Interest

The authors declare that the research was conducted in the absence of any commercial or financial relationships that could be construed as a potential conflict of interest.

## Footnotes

1 http://www.jcat.de/Start.jsp

## Supplementary Material

FIGURE S1 Construction map of recombinant yeast expression plasmid pPICZαA-CATH-TP5.
