# Supplementary figures and images for: In vitro Impact of Yeast Expressed Hybrid Peptide CATH-2TP5 as a Prophylactic Measure Toward Sepsis and Inflammation
Source: Front Bioeng Biotechnol. 2020 Jun 3;8:454. doi: 10.3389/fbioe.2020.00454 (PMC7283555; doi:10.3389/fbioe.2020.00454)

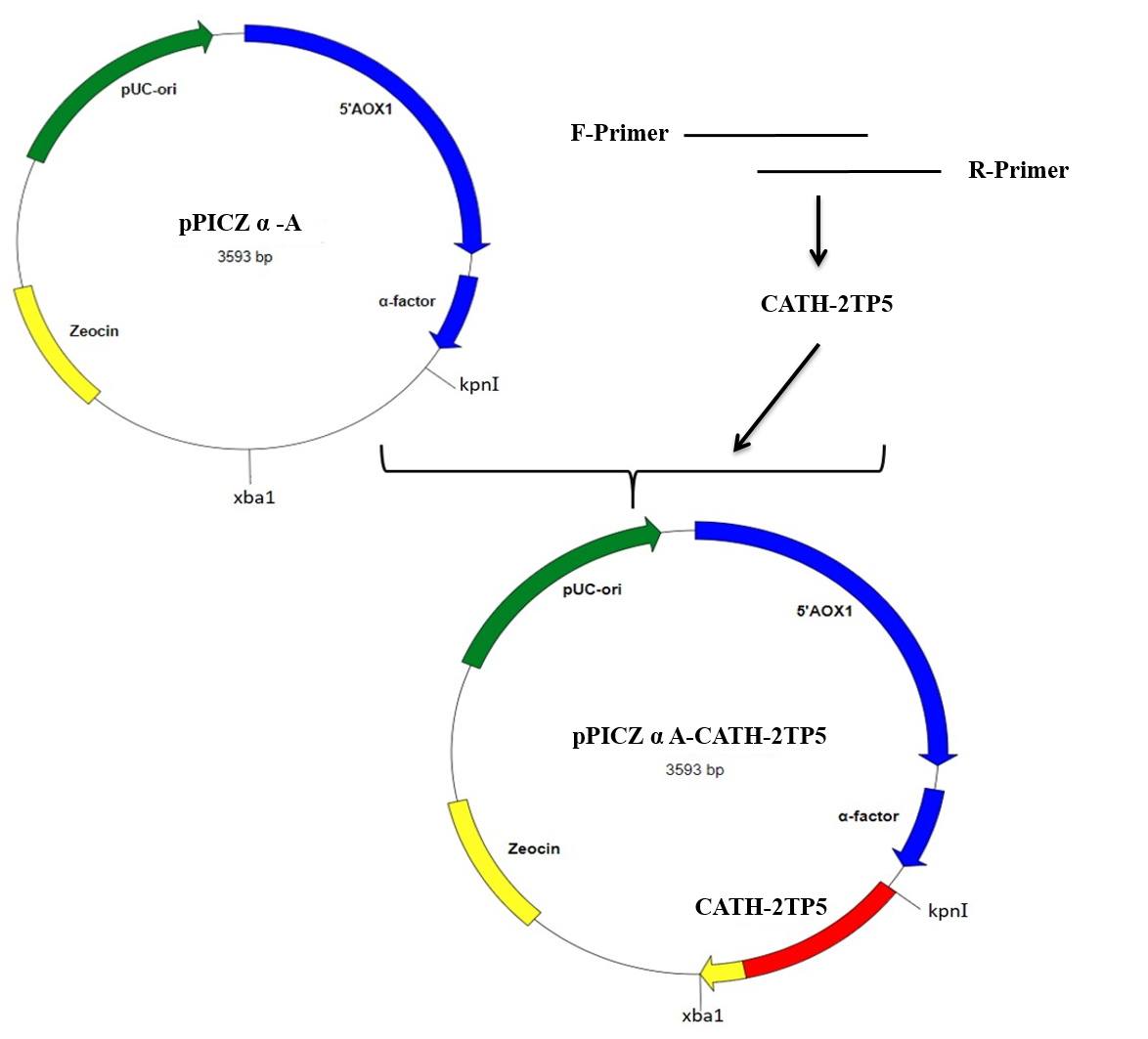

Supplement: FIGURE S1 — Construction map of recombinant yeast expression plasmid pPICZαA-CATH-TP5. [file Image_1.tif]
